# Regulation of bombesin-stimulated cyclooxygenase-2 expression in prostate cancer cells

**DOI:** 10.1186/1471-2199-12-29

**Published:** 2011-07-11

**Authors:** Xiaodong Wen, Celia Chao, Kirk Ives, Mark R Hellmich

**Affiliations:** 1 Department of Surgery, Univ. of Texas Medical Branch, 301 Univ. Blvd., Galveston, TX 77555, USA; 2 Neuroscience and Cell Biology, Univ. of Texas Medical Branch, 301 Univ. Blvd., Galveston, TX 77555, USA; 3 Sealy Center for Cancer Cell Biology, Univ. of Texas Medical Branch, 301 Univ. Blvd., Galveston, TX 77555, USA

**Keywords:** gastrin-releasing peptide receptor, signal transduction, prostate cancer, neuroendocrine differentiation, hormone-refractory

## Abstract

**Background:**

Cyclooxygenase-2 (COX-2) and the bombesin (BBS)-like peptide, gastrin-releasing peptide (GRP), have been implicated in the progression of hormone-refractory prostate cancer; however, a mechanistic link between the bioactive peptide and COX-2 expression in prostate cells has not been made.

**Results:**

We report that BBS stimulates COX-2 mRNA and protein expression, and the release of prostaglandin E_2 _from the GRP receptor (GRPR)-positive, androgen-insensitive prostate cancer cell line, PC-3. BBS-stimulated COX-2 expression is mediated, in part, by p38^MAPK ^and PI3 kinase (PI3K)/Akt pathways, and blocked by a GRPR antagonist. The PI3K/Akt pathway couples GRPR to the transcription factor, activator protein-1 (AP-1), and enhanced COX-2 promoter activity. Although BBS stimulates nuclear factor-kappaB (NF-κB) in PC-3, NF-κB does not regulate GRPR-mediated COX-2 expression. The p38^MAPK ^pathway increases BBS-stimulated COX-2 expression by slowing the degradation of COX-2 mRNA. Expression of recombinant GRPR in the androgen-sensitive cell line LNCaP is sufficient to confer BBS-stimulated COX-2 expression via the p38^MAPK ^and PI3K/Akt pathways.

**Conclusions:**

Our study establishes a mechanistic link between GRPR activation and enhanced COX-2 expression in prostate cancer cell lines, and suggests that inhibiting GRPR may, in the future, provide an effective therapeutic alternative to non-steroidal anti-inflammatory drugs for inhibiting COX-2 in patients with recurrent prostate cancer.

## Background

Prostate cancer is the most commonly diagnosed form of cancer among men in the United States and second only to lung cancer as a cause of cancer-related death. In 2010, the American Cancer Society estimates that over 217,000 new cases of prostate cancer will be diagnosed and more than 32,000 men will die, most from metastatic, androgen (hormone)-refractory disease (American Cancer Society. *Cancer Facts &*Figures 2010, Atlanta: American Cancer Society; 2010; http://www.cancer.org).

Hormone-refractory prostate cancer is characterized, in part, by focal expansion of a malignant cell subpopulation with neuroendocrine (NE) features. NE cells lack expression of androgen receptors, express NE markers, such as neuron-specific enolase and chromogranin A, and contain numerous secretory granules rich in neuropeptides including calcitonin, calcitonin gene-related peptide [1], parathyroid hormone-related protein [2], and the bombesin (BBS)-like peptide, gastrin-releasing peptide (GRP). Although the impact of NE differentiation on poor prognosis and androgen independence has been extensively studied [3], the molecular mechanisms linking NE tumor cells and their bioactive neuropeptides to disease progression are still unclear.

Increased expression of cyclooxygenase-2 (COX-2), an enzyme that catalyzes the synthesis of prostanoids such as prostaglandin E_2 _(PGE_2_) from arachidonic acid [4-6], was identified as an independent predictor of prostate cancer progression [7]. Clinical trials using COX-2 inhibitors in patients with biochemical recurrence of prostate cancer have suggested that COX-2 inhibition may improve survival [8,9], and pre-clinical studies with cell lines and animal models have established a functional link between COX-2 expression and an aggressive cancer phenotype. Specifically, Dandekar and coworkers [10] have demonstrated that overexpression of COX-2 in human prostate cancer cell lines induced chemotherapeutic resistance, decreased apoptosis, and increased tumor angiogenesis and growth. In a transgenic mouse model of prostate carcinogenesis, pharmacological inhibitors of COX-2 suppressed tumor growth and decreased metastatic spread [11,12]. Together, these studies implicate COX-2 in prostate cancer progression; however, the molecular mechanisms leading to its increased expression and the relationship between enhanced expression and NE differentiation requires further investigation.

COX-2 expression can be induced by multiple factors including growth factors, proinflammatory cytokines, and peptide hormones [13-15]. BBS is a 14-amino acid peptide originally isolated from the skin of the frog, *Bombina bombina*, and is a functional homologue to GRP. In humans, GRP binds with high affinity to the GRP receptor (GRPR), a member of the G protein-coupled receptor superfamily [16]. Clinical, histological, and experimental observations have implicated GRP and GRPR in the pathophysiology of prostate cancer progression. Logothetis and Hoosein [17] reported that 40% of patients with hormone-refractory prostate cancer had significantly elevated levels of GRP in their serum. GRP and GRPR are expressed by NE cells in prostate cancer tissue and by prostate cancer-derived cell lines [18,19]; BBS stimulates the growth of both orthotopic and ectopic prostate cancer cell xenografts in athymic nude mice through GRPR-mediated mechanisms [20,21]. BBS also promotes expression of metalloproteinases [22] and increases prostate cancer cell migration and invasion [23-25]. Previously, we reported that BBS stimulates the expression of the proangiogenic genes IL-8 and vascular endothelial growth factor (VEGF) in human prostate cancer cell lines [26].

Since COX-2 and GRPR both regulate cellular processes that contribute to the progression and metastatic spread of prostate cancers and, because BBS has been shown to regulate COX-2 expression in cells from other tissues [27-29], we reasoned that GRPR activation and COX-2 expression may be mechanistically linked in prostate cancer cells. Here, we report that BBS stimulates an increase in COX-2 mRNA, protein expression, and the release of PGE_2 _from the GRPR-positive, androgen-insensitive prostate cancer cell line, PC-3. The stimulatory effects of BBS on COX-2 expression and PGE_2 _production are mediated by p38^MAPK ^and PI3 kinase (PI3K)/Akt pathways and blocked by the selective GRPR antagonist BIM26226. The PI3K/Akt pathway couples GRPR to the activation of the transcription factor, activator protein-1 (AP-1), and enhances COX-2 promoter activity. BBS also stimulates nuclear factor-kappaB (NF-κB) activation in PC-3; however, NF-κB does not regulate GRPR-mediated COX-2 expression. The p38^MAPK ^pathway increases BBS-stimulated COX-2 expression by slowing the degradation of COX-2 mRNA. Expression of recombinant GRPR in the GRPR-negative, androgen-sensitive cell line LNCaP, is sufficient to confer BBS-stimulated COX-2 expression via the p38^MAPK ^and PI3K/Akt pathways. Together, these results define a molecular mechanism for enhanced COX-2 expression in prostate cancer cells, and suggest a means by which NE-differentiated tumor cells and their bioactive neuropeptides may contribute to disease progression.

## Results

### BBS stimulates COX-2 mRNA and protein expression

**Figure 1:**
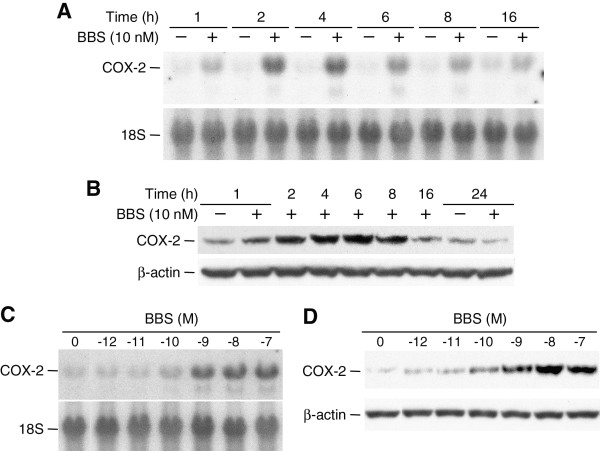
**BBS treatment induces a time- and dose-dependent increase in COX-2 mRNA and protein expression**. A) Autoradiography of Northern blots demonstrating time-dependent increase in COX-2 mRNA steady-state levels in PC-3 cells treated with 10 nM of BBS. Blots were reprobed for 18S ribosomal RNA to ensure equivalent loading and transfer of total RNA in each lane. B) Immunoblot showing time-dependent increase in COX-2 protein levels following treatment with BBS (10 nM). Blots were reprobed for β-actin to ensure the equivalent loading and transfer of protein samples. C, D) Effects of a 4-h treatment with different concentrations of BBS on COX-2 mRNA (C) and protein (D) expression.

To determine whether BBS stimulates COX-2 expression, we treated the androgen-insensitive prostate cancer cell line PC-3 with BBS and measured steady-state levels of COX-2 mRNA and protein at various time points. Compared to vehicle-treated [phosphate buffered saline (PBS)] control cultures, COX-2 mRNA was increased at 1 h following addition of BBS (10 nM) and peaked between 2 and 6 h (Figure 1A). Increased COX-2 protein was also detected at 1 h following BBS treatment, peaked between 4 and 8 h, and returned to baseline levels by 24 h (Figure 1B). Consistent with the lack of change in basal COX-2 mRNA levels over the time course (Figure 1A); we did not observe a change in the basal expression of COX-2 protein in non-treated cells (data not shown). Induction of both COX-2 mRNA and protein expression was dependent on the concentration of BBS. Increased COX-2 mRNA and protein levels were detected in cells treated with as little as 0.1 nM BBS for 4 h and maximal induction was observed in cells treated with 1 to 10 nM BBS (Figure 1C and 1D, respectively).

### GRPR mediates BBS-stimulated COX-2 protein expression and PGE_2 _synthesis

**Figure 2:**
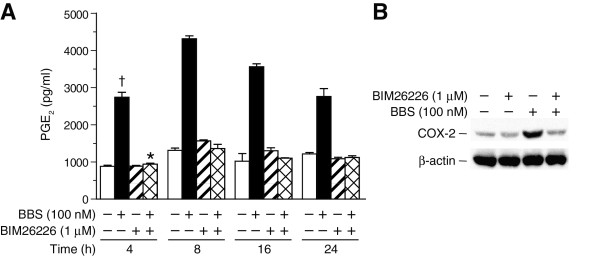
**GRP receptor mediates BBS-stimulated PGE_2 _release and COX-2 expression**. A) PC-3 cells were pre-treated with BIM26226 (1 μM) for 30 min and then incubated with or without 10 nM of BBS over a time course. The amount of PGE_2 _released into the culture media was assessed by enzyme-linked immunoassay (Methods). The data was normalized to total cellular protein per well. [† BBS vs. vehicle (no treatment), p < 0.01; * BBS+BIM26226 vs. BBS alone, p < 0.01]. B) Immunoblot showing the effects of BIM26226 on BBS-stimulated COX-2 protein expression. PC-3 cells were pretreated with BIM26226 for 30 min and then treated with BBS for 4 h. Blots were reprobed for β-actin to ensure the equivalent loading and transfer of protein samples.

COX-2 converts arachidonic acid, released from phospholipids by the action of phospholipase A_2_, to prostaglandin H_2 _- the common precursor of all prostaglandins, including PGE_2_. To assess whether BBS stimulation of COX-2 expression was associated with an increased prostaglandin synthesis, the levels of PGE_2 _released from PC-3 cells were measured using an enzyme-linked immunosorbent assay. Compared to vehicle-treated control cultures, BBS induced a time-dependent increase in the PGE_2 _levels that peaked between 4 and 16 h (Figure 2A, open bar vs. black bar, † p ≤ 0.01). Pretreatment with the GRPR-selective antagonist, BIM26226 ([D-F_5_-Phe^6^, D-Ala^11^]Bombesin(6-13)Ome] [30], (1 μM) for 30 min blocked the BBS-induced increase in PGE_2 _(Figure 2A, black bar vs. cross-hatched bar), *p ≤ 0.01). Consistent with this observation, BIM26226 also inhibited BBS-stimulated increases in COX-2 protein expression (Figure 2B).

### BBS activates multiple intracellular signaling pathways in PC-3 cells

**Figure 3:**
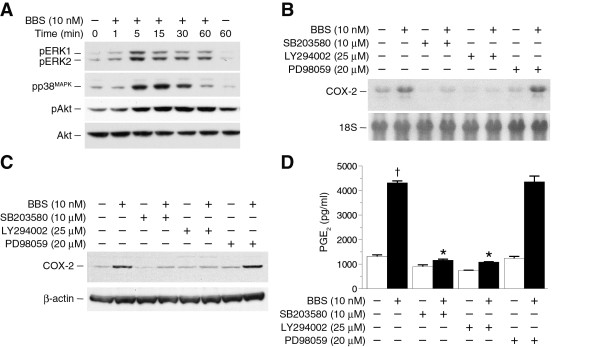
**The PI3K/Akt and p38^MAPK ^pathways mediate BBS-stimulated COX-2 expression and PGE_2 _synthesis**. A) PC-3 cells were treated with 10 nM BBS over a time course, and the levels of phosphorylation (activated) of ERK1 and ERK2 (pERK1 and pERK2), p38^MAPK ^(pp38^MAPK^) and Akt (pAkt) were detected by immunoblotting with phospho-specific antibodies. The level of total Akt was assessed to insure the equivalent loading of protein samples in each lane. B, C) Effects of the p38^MAPK ^inhibitor SB203580, the PI3K/Akt inhibitor, LY294002, and the ERK inhibitor, PD98059 on BBS-stimulated COX-2 mRNA (B) and protein (C) expression. Blots were reprobed for 18S ribosomal or β-actin to ensure the equivalent loading and transfer of RNA and protein samples, respectively. D) Effects of the SB203580, LY294002, PD98059 on BBS-stimulated (6 h) PGE_2 _elaboration from PC-3 cells [† BBS vs. vehicle, p ≤ 0.01; * BBS+SB203580 or LY294002 vs. BBS alone, p ≤ 0.01, n = 3].

COX-2 expression is regulated by multiple intracellular signaling pathways including the MAPK pathways, MEK/ERK and p38^MAPK^, and the PI3K/Akt pathway [31]. To determine if these pathways were activated by BBS, PC-3 cells were treated with peptides over a time course and the activation state of each pathway assessed by immunoblotting. BBS treatment (10 nM) induced a time-dependent increase in the levels of activated (phosphorylated) ERK1 (pERK1) and ERK2 (pERK2) (Figure 3A). The levels increased rapidly with a peak phosphorylation occurring between 1 and 15 min and remained elevated above baseline 60 min after treatment with BBS (Figure 3A). BBS stimulated a transient activation of p38^MAPK^. The levels of phospho-p38^MAPK ^(pp38^MAPK^) peaked between 5 and 30 min and, in contrast to the activation of either ERK1 and 2 or Akt, returned to baseline levels by 60 min (Figure 3A). The levels of phospho-Akt (pAkt) increased at 5 min, reached a plateau by 15 min, and remained elevated for the duration of the time course (Figure 3A). Over the same time course, we did not observe any change in the activation state of these pathways in non-stimulated cells (data not shown).

### BBS-stimulated COX-2 expression is regulated by the p38^MAPK ^and PI3K/Akt pathways, but not the MEK/ERK signaling axis

To assess the roles of the p38^MAPK^, PI3K/AKt, and MEK/ERK pathways in BBS-stimulated COX-2 expression, PC-3 cells were pretreated (30 min) with either the p38^MAPK ^inhibitor (SB203580, 10 μM), the PI3K inhibitor (LY294002, 25 μM) or the MEK1,2 inhibitor (PD98059, 20 μM) and then stimulated with BBS (10 nM) for 4 h. Agonist treatment failed to increase either COX-2 mRNA or protein levels when the cells were pretreated with either SB203580 or LY294002 (Figure 3B and 3C). In contrast, pretreatment with PD98059 did not inhibit BBS-stimulated increases in COX-2 mRNA nor protein expression. Consistent with these observations, SB203580 or LY294002 inhibited BBS-stimulated PGE_2 _release from PC-3 cells, whereas PD98059 had no effect (Figure 3D).

### Inhibition of PI3K/Akt pathway reduces BBS-stimulated COX-2 promoter activity

**Figure 4:**
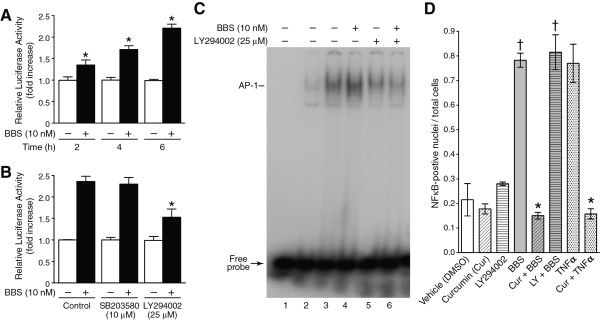
**The PI3K/Akt pathway regulates BBS-stimulated COX-2 promoter activity and AP-1 activation**. PC-3 cells were co-transfected with plasmids containing the 1.4 kb human COX-2 promoter 5' of a luciferase reporter gene and β-galactosidase. A) Twenty-four hours after transfection, cells were treated either with vehicle or 10 nM BBS for 2, 4, and 6 h. Changes in luciferase activity (mean ± S.D, n = 3) are expressed relative to vehicle treated at each time point after normalizing for transfection efficiency using β-galactosidase activity [* BBS vs. time-point matched vehicle control, p < 0.05]. B) Pre-treatment with LY294002 (25 μM) partially inhibited BBS-stimulated COX-2 promoter activity [* LY294002 vs. BBS alone, p < 0.05, n = 3], whereas SB203580 (10 μM) had no effect. C) Autoradiogram showing binding of nuclear proteins to ^32^P-labeled oligonucleotide containing the AP-1 consensus sequence [lane 1, radiolabeled probe only; lane 2, nuclear proteins + radio-labeled probe and excess unlabeled probe; lane 3, nuclear protein from vehicle-treated cells; lane 4 nuclear proteins from cells treated with BBS (10 nM) for 30 min; lanes 5 and 6, nuclear proteins from cells pretreated with LY294002 (25 μM) for 30 min followed by vehicle or BBS for 30 min]. D) PC-3 cells were treated with BBS (10 nM) or TNF-α (10 ng/ml) alone or in combination with inhibitors for 30 min, fixed, and immunostained with an antibody to the p65 subunit of NF-κB. Quantification of the effects of curcumin (Cur) (20 μM) and LY294002 (LY) (25 μM) on BBS-stimulated NF-κB nuclear translocation. Data are expressed as the ratio of NF-κB-positive stained nuclei divided by the total number of cells per high power field (200X). Four fields were counted for each condition from 3 independent experiment [† BBS or BBS + LY294002 vs. vehicle or LY294002 alone, p ≤ 0.001; * BBS + curcumin vs. BBS alone or TNFα + curcumin vs. TNFα alone, p ≤ 0.001].

The cellular levels of COX-2 mRNA can be regulated both by enhanced gene transcription and inhibition of message degradation [31,32]. To determine whether BBS treatment enhanced COX-2 gene transcription, PC-3 cells were first transiently transfected with a transcription reporter construct consisting of 1.4 kb of the human COX-2 promoter coupled to a luciferase gene and then stimulated with BBS over a time course. BBS induced a time-dependent increase (1.4- to 2.3-fold) in COX-2 promoter activity when compared to vehicle-treated control cell cultures (Figure 4A). To determine whether the p38^MAPK ^or PI3K/Akt pathways were involved in BBS-stimulated COX-2 transcription, cells were pretreated with SB203580 (10 μM) or LY294002 (25 μM) for 30 min followed by a 6-h treatment with BBS (10 nM). Compared to BBS treatment alone, LY294002 inhibited approximately 50% of the increase in BBS-stimulated luciferase activity (Figure 4B). In contrast, SB203580 had no effect on BBS-stimulated COX-2 promoter activity (Figure 4B), suggesting that the PI3K/Akt pathway, not the p38^MAPK ^pathway, is involved in BBS-induced COX-2 gene transcription in PC-3 cells.

### LY294002 inhibits BBS-stimulated AP-1 binding activity but not NF-κB nuclear translocation

The human COX-2 promoter contains multiple regulatory sites that bind transcription factors including nuclear factor-κB (NF-κB) [33,34] and AP-1 [35]. Electrophoresis mobility shift assays showed that BBS treatment of PC-3 cells induced an increase in AP-1 binding activity (Figure 4C) that was inhibited by pretreatment with LY294002 (25 μM) for 30 min (Figure 4C), suggesting that PI3K/Akt-mediated AP-1 activation is involved in BBS regulation of the COX-2 promoter activity. In addition to activation of AP-1, we previously reported that BBS also induced NF-κB activation in PC-3 cells [26]. NF-κB is an inducible dimeric transcription factor that belongs to the Rel/NF-κB family of proteins [36]. Activation of NF-κB involves its dissociation from the inhibitor protein, IκB, followed by its translocation to the nucleus where it binds to specific DNA sequences in the promoter regions of multiple genes including COX-2 [37]. To confirm that BBS activated NF-κB, PC-3 cells were treated with peptide for 30 min, fixed, and immunostained with an antibody to the p65 subunit of NF-κB. Treated cells demonstrated increased nuclear NF-κB immunoreactivity when compared with vehicle-treated cultures (Figure 4D). Pretreating cells with the NF-κB inhibitor, curcumin (diferuloylmethane, 20 μM), inhibited both BBS- and TNF-α-induced NF-κB translocation to the nucleus (Figure 4D). To assess the role of the PI3K/Akt pathway in BBS-induced NF-κB activation, cells were pretreated with LY294002 for 30 min, followed by 30 min treatment with agonist. LY294002 had no effect on the BBS-induced NF-κB translocation (Figure 4D). Since LY294002 inhibits COX-2 mRNA expression (Figure 3A), COX-2 promoter activity (Figure 4B) and AP-1 binding (Figure 4C), these data suggest that AP-1 and not NF-κB regulates BBS-stimulated COX-2 promoter, in part, through activation of the PI3K/Akt pathway.

### p38^MAPK ^activity enhances the stability of COX-2 mRNA

**Figure 5:**
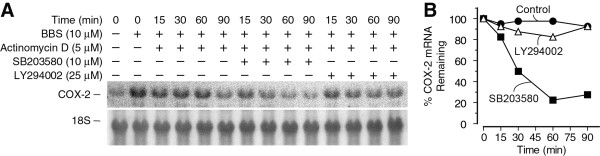
**p38^MAPK ^activity enhances COX-2 mRNA stability**. A) Autoradiogram of Northern blot showing the effects of p38^MAPK ^and PI3K/Akt inhibition on COX-2 mRNA degradation. Cells were stimulated with BBS for 4 h, and attenuated 4 μM actinomycin D was added (t = 0) alone or with SB203580 or LY294002 and total RNA isolated at 15, 30, 60, and 90 min. Blots were reprobed for 18S ribosomal RNA to ensure equivalent loading and transfer of total RNA in each lane. B) Graph of densitometric analysis of blot in panel A.

Since inhibition of the p38^MAPK ^pathway did not block BBS-stimulated COX-2 promoter activity (Figure 4B), we assessed its potential role in regulating COX-2 mRNA stability. PC-3 cells were stimulated for 4 h with BBS (10 μM) and then treated with actinomycin D (5 μM) alone or in combination with either SB203580 or LY294002. RNA was isolated from the cells over a time course and the level of COX-2 mRNA at each time point assessed by Northern blotting (Figure 5A). COX-2 mRNA degraded slowly in BBS-stimulated cells treated with actinomycin D alone or in combination with LY294002; less than 20% of the COX-2 message was lost over the 90-min time course (Figure 5A and 5B). In contrast, the levels of COX-2 mRNA decreased rapidly in cells treated with a combination of actinomycin D and SB203580; approximately 50% of the COX-2 message was degraded in cells treated for 30 min (Figure 5B), suggesting that p38^MAPK ^activity stabilizes BBS-induced increases in COX-2 mRNA levels.

### Expression of GRPR is sufficient to confer BBS-inducible COX-2 expression in LNCaP cells

**Figure 6:**
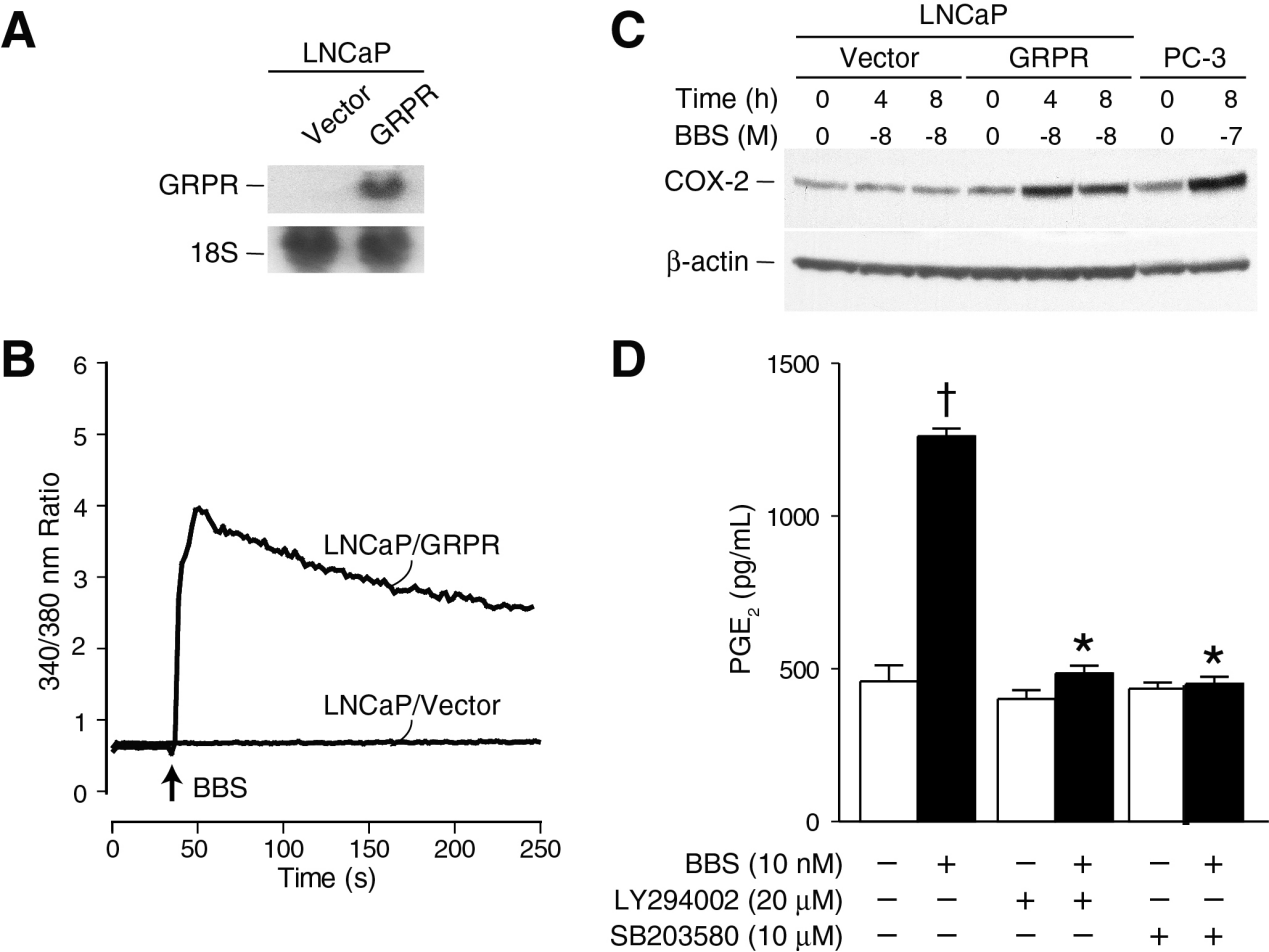
**Expression of GRPR in LNCaP cells confers BBS-stimulated COX-2 expression and PGE_2 _synthesis**. A) Northern blot confirming the expression of GRPR in transfected LNCaP cells (n = 1). B) Time-dependent change in intracellular Ca^2+ ^concentration measured with Fura-2; comparison of GRPR transfected (LNCaP/GRPR) to control vector-transfected LNCaP cells (LNCaP/Vector) (tracing represents the average change in [Ca^2+^]_i _from 40 cells). C) Immunoblot showing effects of BBS treatment on COX-2 protein expression in LNCaP/Vector and LNCaP/GRPR cells. PC-3 cells were used as a positive control. Blots were reprobed for β-actin to ensure the equivalent loading and transfer of protein samples. D) Effects of SB203580 and LY294002 on BBS-stimulated PGE_2 _elaboration form LNCaP/GRPR cells. Cells were pretreated for 30 min with the inhibitors and then stimulated with BBS for 6 h. PGE_2 _levels in the culture media were determined as described (Material and Methods) [† BBS vs. vehicle, p < 0.01; * BBS+SB203580 or LY294002 vs. BBS alone, p < 0.01].

LNCaP prostate cancer cells, in contrast to the PC-3 cells, are androgen-responsive, poorly invasive [23] and do not have specific GRPR binding sites, as assessed by radioligand binding to ^125^I[Tyr4]-Bombesin [38] and [Ca^2+^]_i _mobilization in response to BBS [39]. To determine whether the acquisition of the GRPR was sufficient to confer BBS-inducible COX-2 expression, LNCaP cells were stably transfected with an expression plasmid containing GRPR cDNA downstream of the constitutively-active CMV promoter [40]. GRPR mRNA expression was confirmed by Northern blot (Figure 6A), and the functional status of the receptor was demonstrated by intracellular Ca^2+ ^imaging using Fura-2 (Figure 6B). Immunoblots revealed that BBS stimulated COX-2 protein expression in GRPR-transfected cells, but had no effect on LNCaP cells expressing control vector (Figure 6C). Also, similar to PC-3 cells, pretreatment of GRPR-transfected LNCaP cells with either SB203580 (10 μM) or LY294002 (25 μM) completely inhibited BBS-stimulated PGE_2 _release, suggesting that GRPR regulates COX-2 activity via the p38^MAPK ^and PI3K/Akt pathways (Figure 6D).

## Discussion

Although the aberrant overexpression of COX-2, the BBS-like peptide, GRP, and GRPR has been documented for prostate cancers with NE features, particularly in the setting of recurrent disease [3,7,41], a mechanistic link between GRPR activation and COX-2 expression in prostate cancer cells has not been made. The studies presented here establish that BBS can stimulate COX-2 expression and begins to define the molecular signal transduction pathways linking GRPR to COX-2.

Expression of COX-2 can be regulated by multiple signaling pathways that affect both gene transcription and post-transcriptional mRNA processing [42-45]. We found that BBS-stimulated COX-2 expression requires the activation of both the p38^MAPK ^and PI3K/Akt pathways in PC-3 and LNCaP cells expressing recombinant GRPR. LY294002 partially inhibited BBS-stimulated COX-2 promoter activity, decreased COX-2 mRNA and protein levels, and reduced PGE_2 _secretion, whereas, inhibition of p38^MAPK ^destabilized BBS-stimulated COX-2 mRNA, resulting in a decrease of both COX-2 protein expression and PGE_2 _production. The MEK inhibitor, PD98059, had no effect on either BBS-stimulated COX-2 expression or PGE_2 _secretion, in contrast to previously published data from our laboratory showing that, in intestinal epithelial cells, BBS stimulated COX-2 expression through a MEK/ERK-dependent pathway [40].

Sequence analysis of the 5'-flanking region of the human COX-2 gene has identified multiple potential transcriptional regulatory elements including two NF-κB sites located at 213 to 222 and 438 to 447 [46,47] base pairs 5' of the transcriptional start site, and an AP-1 site located ~60 base pairs upstream from the start site [31]. Although we have previously reported NF-κB-dependent regulation of VEGF and IL-8 expression [26] in PC-3 cells, in the present study NF-κB does not appear to be involved in GRPR-regulation of the endogenous COX-2 promoter. This conclusion is based on the observation that LY294002 can inhibit COX-2 mRNA expression and partially reduced promoter activity, but does not block BBS-stimulated NF-κB activation. In contrast, LY294002 does inhibit BBS-induced AP-1 binding, suggesting that AP-1 may be the primary transcription factor involved in BBS-stimulated COX-2 mRNA expression in PC-3 cells. These findings are consistent with data from intestinal epithelial cells showing that BBS-stimulated COX-2 expression via an AP-1-dependent pathway [40].

Despite the fact that more than 80% of patients with late stage prostate cancer initially respond to androgen-ablation therapy [48], more than half will progress to a hormone-refractory disease within 16 to 18 months after treatment [49]. Elucidation the molecular mechanisms mediating the conversion of androgen-sensitive prostate cancers to hormone-refractory disease is one of the most critical tasks in improving current treatment strategies and patient survival. A recent study evaluating archival prostate cancer specimens by immunohistochemical methods identified COX-2 levels as an independent predictor of prostate cancer recurrence [7]. At 62-months follow-up, COX-2 immunostaining predicted cancer progression with 82% sensitivity and 81% specificity. Additionally, two clinical trials have generated early, but promising, data that inhibition of COX-2 can benefit patients with biochemical recurrence of prostate cancer (i.e., the same transcription factor can regulate increasing PSA levels, but no clinical evidence of disease following androgen-ablation therapy). In the first study, Pruthi and associates [8] studied 12 patients who were given the selective COX-2 inhibitor, celecoxib, following a diagnosis of biochemical recurrence. Five of the 12 patients had decreased PSA levels, and three of the 12 had stabilization of their PSA levels at 3, 6, and 12 months following the initiation of therapy. In a second larger randomized control trial comparing placebo (n = 40) to celecoxib (n = 38), the group receiving celecoxib for 6 months showed a significantly decreased rate of rise in PSA levels when compared to the placebo control patients [9]. Together, these trials suggest that targeting COX-2 can be beneficial. Unfortunately, the studies were terminated by the United States Food and Drug Administration because of the potential risk of cardiovascular complications with the current cadre of COX-2 inhibitors.

Novel strategies for inhibiting COX-2 could once again make it a viable therapeutic target in the future. Inhibiting GRPR may provide an effective therapeutic alternative for decreasing COX-2 expression and activity in patients with recurrent prostate cancer. Proof of this concept is provided by a recent pre-clinical study that evaluated the effects of the GRPR antagonist, RC-3940-II, in an orthotopic non-small-cell lung carcinoma model [29]. Similar to prostate cancers, lung cancers express GRP and GRPR [50] where they promote tumor progression and metastatic spread through autocrine and paracrine mechanisms. Hohla and colleagues [29] showed that daily treatment of NSCLC tumor-bearing mice with RC-3940-II reduced the mean lung tumor weight by up to 53%. Importantly, the decreased tumor growth was associated with antagonist-induced decreases in p-Akt levels and COX-2 expression suggesting, together with the data presented herein, that GRPR blockage may be an effective means of decreasing COX-2 expression within receptor-positive tumor tissue.

## Conclusion

Our study establishes a mechanistic link between GRPR activation and enhanced COX-2 expression in prostate cancer cell lines, and suggests that inhibiting GRPR may provide an alternative to non-steroidal anti-inflammatory drugs for inhibiting COX-2 in patients with recurrent prostate cancer.

## Methods

### Materials

BBS was purchased from Bachem (Torrance, CA). Inhibitors of p38^MAPK ^(SB203580), MEK-1 and -2 (PD98059), and PI3-kinase (PI3K) (LY294002) were purchased from CalBioChem (San Diego, CA). The antibody to phospho-p38^MAPK ^was purchased from Promega (Madison, WI). Antibodies to ERK-1 and -2, phospho-ERK1 and ERK2, p38^MAPK^, and human COX-2 antibody were obtained from Santa Cruz (Santa Cruz, CA). Antibodies to Akt and phosphor-Akt were purchased from Cell Signaling Technology, Inc. (Beverly, MA). The β-actin antibody was purchased from SIGMA (St. Louis, MO). Arachidonic acid was purchased from Cayman Chemical (Ann Arbor, MI). GRPR expression vector was a gift from Dr. James F. Battey (National Institutes of Health, Bethesda, MD).

### Cell culture

The PC-3 and LNCaP cell lines were obtained from American Type Culture Collection (Manassas, VA) and cultured at 37°C in RPMI 1640 supplement with L-glutamine, 10% heat-inactivated fetal bovine serum (FBS) (Hyclone Laboratories Inc., Logan, UT) and 1 mM sodium pyruvate (Sigma-Aldrich Corp., St. Louis, MO). For all experiments, the cells were cultured in serum-free media for 18-24 h prior to treatments.

### RNA isolation and Northern blot analysis

Cells (1.5 × 10^6^) were plated in 100 mm tissue culture dishes. After 24 h, the cells were serum starved for an additional 20 h and then treated as described in the figure legends. RNA was isolated using ULTRASPEC™RNA Isolation System (Biotecx, Houston, TX), resolved on 1% agarose/formaldehyde gels, and transferred onto Hybond-N+ membrane (Amersham Biosciences Corp, Piscataway, NJ). The membrane was hybridized with human COX-2 cDNA probe labeled with [α-^32^P]dATP (Perkin Elmer Life Sciences Inc., Boston, MA) using a random-priming DNA-labeling kit (Stratagene, Cedar Creek, TX). The specific hybridization was visualized by autoradiography. The membrane was re-hybridized with a probe for 18S ribosomal RNA to confirm RNA integrity and equivalent loading of each sample. With the exception of the Figure 6A, all RNA isolations and Northern blots were repeated a minimum of three times.

### Western blot analysis

Cells were incubated in serum-free media for the indicated period of time, treated with peptide hormone and/or drug as described in the figure legends and lysed using a solution containing 50 mM Tris (pH 7.4), 150 mM NaCl, 1 mM EDTA, 1% Nonidet P-40, 1 mM sodium orthovanadate, 5 mM β-glycerophosphate, 10 mM sodium pyrophosphate, 1 mM NaF, 1 mM PMSF, and 1 Protease Inhibitor Cocktail Tablet/50 ml (Roche Applied Science, Indianapolis, IN). The detergent-insoluble cellular material was removed by centrifugation at 14000 rpm for 15 min, and the protein concentration of the supernatant was determined using BioRad Dye Reagent Concentrate (BioRad Laboratories, Inc., Hercules, CA). Proteins (40-100 μg/lane) were resolved on 4-12% continuous gradient Bis-Tris-HCl buffered polyacrylamide gels (Invitrogen, Carlsbad, CA), transferred to a PVDF membrane (Millipore Corporation, Bedford, MA), and blocked with 5% non-fat milk. Membranes were probed with the primary antibodies at dilutions indicated in the figure legends. Specific immunoreactive bands were detected using enhanced chemiluminescence (ECL)™ Western Blotting Detection Reagent (Amersham Biosciences Corp, Piscataway, NJ) and Kodak X-Omat film. All protein isolations and Western blots were repeated a minimum of three times.

### PGE_2 _assay

Cells (1.2 × 10^5^/well) were seeded in 12-well plates for 24 h. After culturing for 20 h in serum-free media, the cells were treated with BBS for the period of 4, 8, 10, 16, and 24 h. Arachidonic acid (15 μM) was added 30 min prior to the collection of media. Culture media (100 μl) from each well were analyzed for PGE_2 _by using Biotrak Enzyme immunoassay (EIA) system (Amersham Biosciences, Piscataway, NJ). All assays where repeat at least three times.

### Luciferase reporter gene assay

Cells (1.2 × 10^5^/well) were seeded into 12-well plates and co-transfected with 250 ng of plasmid DNA containing the human COX-2 promoter coupled to a luciferase reporter gene and 30 ng of plasmid containing β-galactosidase using LipofectAMINE™ Reagent (Invitrogen, Carlsbad, CA). Cells were treated as described in the figure legends. Luciferase and β-galactosidase activity were assayed using Enhanced Luciferase Assay Kit (BD Biosciences, San Diego, CA) and Galacto-Light Plus™ Systems (Applied Biosystems, Bedford, MA), respectively. The transfections and luciferase assays were repeated three times.

### Immunofluorescence microscopy

PC-3 cells were cultured on glass coverslips. Before immunostaining for NF-κB p65 subunit, the cells were treated either with vehicle, curcumin (20 μM), BBS (10 nM), TNF-α (10 ng/ml), or a combination of curcumin and BBS or TNF-α for 30 min at 37°C, fixed with 4% paraformaldehyde (15 min), permeabilized with 0.3% Triton X-100 (10 min), and incubated in blocking solution (1% BSA in PBS; 20 min). After incubating the cells with anti-NF-κB antiserum (1:100 dilution; San Cruz Biotechnology Inc., San Cruz, CA) for 1 h at room temperature, the cells were washed three times with PBS and incubated with a goat anti-rabbit IgG antibody labeled with Alexa 488 (Molecular Probes, Inc., Eugene, OR; 1:2000; 30 min). The immunostaining procedure was repeated at least three times. Specific immunostaining was visualized with a Nikon Eclipse fluorescence microscope.

### Electrophoretic mobility shift assay

Nuclear extracts were prepared as previously described [26]. Oligonucleotides (Promega, Madison, WI) of which the sequence corresponding to the AP-1 binding site consensus sequence (5'- CGC TTG ATG AGT CAG CCG GAA -3') were end-labeled with [γ-^32^P]ATP and T4 polynucleotide kinase respectively. Electrophoretic mobility shift assay (EMSA) reaction mixtures contained 50,000 cpm of ^32^P-end-labeled oligonucleotide, 20 μg of nuclear protein extract, and gel shift binding buffer (Promega) in a final volume of 20 μl. Reaction mixtures were resolved on 4% nondenaturing polyacrylamide gel electrophoresis at 200 V for 2 h. Gels were dried and visualized by autoradiography. Preparation of nuclear extracts and EMSA was performed a minimum of three times.

### Intracellular Ca^2+ ^measurements

Cells, grown on 25-mm glass coverslips, were washed with a physiological medium (KRH) containing NaCl (125 mM), KCl (5 mM), KH_2_PO_4 _(1.2 mM), MgSO_4 _(1.2 mM), CaCl_2 _(2 mM), glucose (6 mM), HEPES (25 mM; pH 7.4), and loaded with 2 μM Fura-2 AM (Molecular Probes, Inc.) for 50 min at 25°C. The cells were treated with BBS (10 nM), and single cell changes in the concentration of free intracellular Ca^2+ ^([Ca^2+^]_i_) were recorded using a Nikon Diaphot inverted microscope (Garden City, NY) and a CCD camera (Dage-MTI, Inc., Michigan City, IN). Data points were collected every 1-8S from ~35 cells/coverslip and processed using ImageMaster software. Data are presented as the mean change in [Ca^2+^]_i_.

### Statistical analysis

Statistical analysis was performed using GraphPad InStat 3.0 (GraphPad Software, Inc.). Statistical significance was assumed if *P *≤ 0.05.

## List of abbreviations

COX-2: Cyclooxygenase-2; BBS: bombesin; GRP: gastrin-releasing peptide; PI3K: PI3 kinase; AP-1: activator protein-1; NF-κB: nuclear factor-kappaB; NE: neuroendocrine; GRPR: GRP receptor; EIA: Enzyme immunoassay

## Authors' contributions

XDW performed the majority of the cellular, molecular, and protein assays. KI performed the calcium and prostaglandin assays. CC participated in the study's design and coordination, statistical analyses, and helped draft the manuscript. MRH conceived the study, participated in its design and coordination, and helped draft the manuscript. All authors read and approved the final manuscript.
